# Structure, Biological Functions, Separation, Properties, and Potential Applications of Milk Fat Globule Membrane (MFGM): A Review

**DOI:** 10.3390/nu16050587

**Published:** 2024-02-21

**Authors:** Chao Nie, Yunyi Zhao, Xifan Wang, Yixuan Li, Bing Fang, Ran Wang, Xiaoyu Wang, Haiping Liao, Gengsheng Li, Pengjie Wang, Rong Liu

**Affiliations:** 1 Key Laboratory of Functional Dairy, Co-Constructed by Ministry of Education and Beijing Government, Department of Nutrition and Health, China Agricultural University, Beijing 100083, China; 2 Food Laboratory of Zhongyuan, Luohe 462000, China; 3 Department of Obstetrics and Gynecology, Columbia University Irving Medical Center, New York, NY 10032, USA

**Keywords:** milk fat globule membrane, phospholipid, separation, emulsifier, applications

## Abstract

Background: The milk fat globule membrane (MFGM) is a thin film that exists within the milk emulsion, suspended on the surface of milk fat globules, and comprises a diverse array of bioactive components. Recent advancements in MFGM research have sparked a growing interest in its biological characteristics and health-related functions. Thorough exploration and utilization of MFGM as a significant bioactive constituent in milk emulsion can profoundly impact human health in a positive manner. Scope and approach: This review comprehensively examines the current progress in understanding the structure, composition, physicochemical properties, methods of separation and purification, and biological activity of MFGM. Additionally, it underscores the vast potential of MFGM in the development of additives and drug delivery systems, with a particular focus on harnessing the surface activity and stability of proteins and phospholipids present on the MFGM for the production of natural emulsifiers and drug encapsulation materials. Key findings and conclusions: MFGM harbors numerous active substances that possess diverse physiological functions, including the promotion of digestion, maintenance of the intestinal mucosal barrier, and facilitation of nerve development. Typically employed as a dietary supplement in infant formula, MFGM’s exceptional surface activity has propelled its advancement toward becoming a natural emulsifier or encapsulation material. This surface activity is primarily derived from the amphiphilicity of polar lipids and the stability exhibited by highly glycosylated proteins.

## 1. Introduction

**Figure 1:**
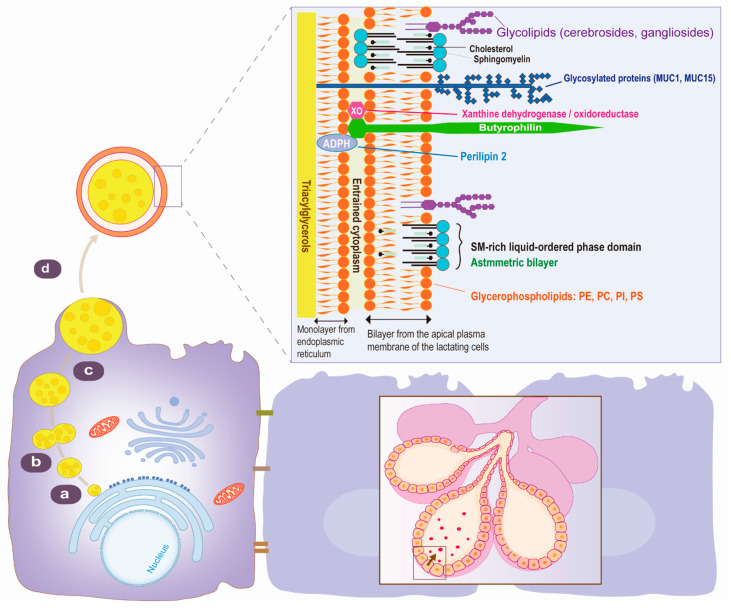
Secretion, structure, and common glycoproteins of MFGM. (a) Endoplasmic reticulum membranes form triacylglycerol-rich droplets. (b) Fusion of fat droplets generates larger fat droplets. (c) Transportation of fat droplets to the apical cell region. (d) Mammary epithelial cells secrete fat droplets through exocytosis, forming a complete membranous structure. The arrow marks milk fat globule (MFG).

The milk fat globule membrane (MFGM) is a nanometer-scale membrane structure present on the surface of milk fat globules in mammalian milk [1]. It comprises various bioactive components, such as glycoproteins, phospholipids, sphingolipids, cholesterol, and free fatty acids (Figure 1), which are crucial for brain development [2]. As scientific research on MFGM deepens, there is a growing interest in understanding its physiological functions and mechanisms of action. The primary function of MFGM is to protect milk fat globules from digestion, maintaining the stability and uniformity of milk emulsion through its proteins and polar lipids [3,4]. This property has led to the development of MFGM-based food emulsifiers [5,6]. Furthermore, MFGM exhibits multiple biological activities, including immunoregulation, promotion of calcium absorption, and enhancement of intellectual development [5,7,8], indicating substantial commercial potential. However, many components of MFGM remain unclear, while effective extraction and separation technologies are yet to be established, impeding further elucidation of its functions and mechanisms.

Currently, the primary application of MFGM is as an ingredient and emulsifier in infant formula [9], aiding in the growth and development of infants while enhancing the digestibility and absorption of milk emulsion, mimicking the composition of breast milk [4,10]. As a consequence, the development of infant formula enriched with MFGM has become a prominent topic. With advancements in MFGM separation and purification technologies, the application of MFGM has expanded to other fields, including food additives and encapsulation materials [11,12].

## 2. Structure and Composition of MFGM

### 2.1. Structure

Milk fat globules consist of a triglyceride core encased by the MFGM. Triglyceride-rich lipid droplets (LDs) originate within the endoplasmic reticulum (ER) membrane. The formation of LDs at the ER is prominently orchestrated by Seipin. As the neutral lipids within the interleaflet space mature, LDs undergo a dynamic process, leading to the detachment or pinching off from the ER membrane into the cytosol. This sophisticated cellular mechanism ensures the controlled maturation of LDs, contributing to lipid homeostasis. The lipid droplets are subsequently transported to the apical membrane [13]. These large droplets are secreted through exocytosis from the apical cytoplasmic membrane of mammary epithelial cells, which is a phospholipid bilayer characteristic of secretory cells, thereby forming a complete membranous structure during secretion [14,15]. The MFGM exhibits a three-layered structure with a thickness ranging from 10 to 50 nm [16], with the inner membrane originating from the endoplasmic reticulum and the outer membrane from the apical plasma membrane bilayer of mammary epithelial cells. BTN in the apical plasma membrane undergoes self-aggregation and is combined with XOR, and these aggregates together with proteins, including PLIN2, on the surface of lipid droplets to facilitate the departure of spherical structures containing lipid droplets from the cell. The presence of cytoplasmic material between the inner coat and the outer double membrane layer leads to the formation of ‘cytoplasmic crescents’ [17]. The primary constituents of the MFGM include polar lipids (glycerophospholipids, sphingomyelin, and glycosphingolipids such as gangliosides), proteins (with a high proportion of glycoproteins and enzymes), neutral lipids, and RNA [13].

### 2.2. Components

The components of MFGM include phospholipids, sphingolipids, and membrane-bound proteins, primarily glycoproteins. MFGM proteins and total lipids constitute more than 90% of its dry weight, accounting for approximately 64% of total lipids and 28% of total proteins [2,18]. However, there were significant differences in the composition of MFGM reported in different studies. For example, the breed, lactation period, feed type, season, and environmental factors may affect the composition of MFGM [19].

### 2.3. MFGM Protein Composition

MFGM contains approximately 40 proteins, with the primary proteins including mucin MUC1, xanthine dehydrogenase/oxidase (XDH/XO), butyrophilin (BTN), cluster of differentiation 36 (CD36), periodic acid–Schiff 6/7 (PAS 6/7), periodic acid–Schiff III (PAS III, also called MUC15), perilipin 2, and fatty acid-binding proteins (FABP) [2,16]. Additionally, certain enzymes are present in small amounts or only in specific species. For instance, carboxyl ester lipase and fatty acid-binding protein (FABP) are only found in human MFGM, while glycosylation-dependent cell adhesion molecule 1 (GlyCAM1) is exclusive to bovine MFGM [20]. However, isolating and identifying all the proteins in MFGM remains challenging. Various purification methods are currently employed, including sulfate fractionation, affinity chromatography, ion exchange chromatography, cesium chloride density gradient centrifugation, and gel electrophoresis. Nevertheless, effective purification methods for many proteins are still being established [20]. Table 1 provides an overview of some identified proteins in MFGM, their functions, and the applied extraction methods.

### 2.4. MFGM Lipid Composition

The lipids present in MFGM can be categorized into polar lipids and neutral lipids. The primary polar lipids found in all mammal species include phosphatidylethanolamine (PE), phosphatidylcholine (PC), phosphatidylserine (PS), phosphatidylinositol (PI), lysophosphatidylcholine (LPC), and sphingomyelin (SM) [33]. Neutral lipids in MFGM mainly include triglycerides, with palmitic acid and oleic acid being the most prominent fatty acids [34]. Phospholipids (PLs) constitute approximately 25% of MFGM components [18]. Additionally, minor constituents of MFGM include glycosphingolipids, which encompass cerebrosides (neutral glycosphingolipids containing uncharged sugars) and gangliosides (acidic glycosphingolipids containing sialic acid) [20] (Figure 1).

## 3. Health Benefits of MFGM as a Bioactive Ingredient

### 3.1. Improvement of the Neurodevelopmental Profile

**Figure 2:**
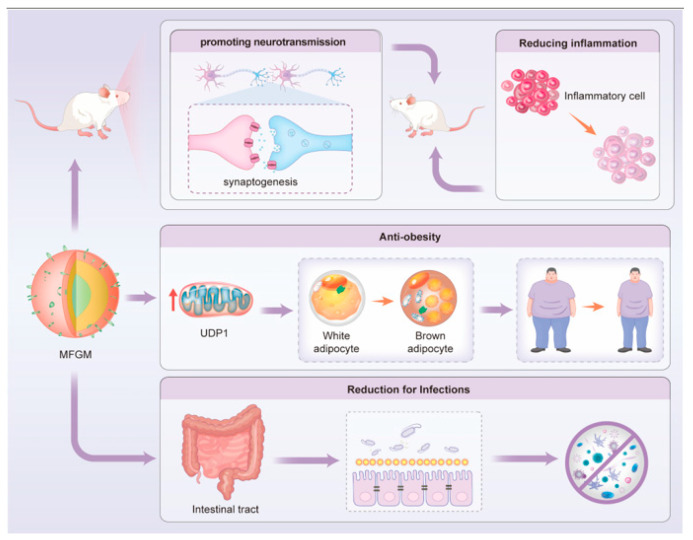
Major in vivo function of MFGM illustrated in a schematic diagram.

MFGM has shown the potential to improve learning and spatial memory abilities in mice by promoting synaptogenesis and neurotransmission (Figure 2) [35]. MFGM was also found to exert neuroprotective effects by reducing neuronal apoptosis and increasing the neuron count in BALB/c mice. In line with these findings, Geng et al. demonstrated that MFGM upregulates genes associated with synaptic morphogenesis (Sorbs2, Rab39, and Cacna1e) and downregulates genes related to neuromodulation (Hp and Lrg1). Differentially expressed proteins may enhance spatial memory and cognition in mice by facilitating synapse formation and increasing the abundance of neurotransmitter receptors [36].

### 3.2. Anti-Inflammatory Activity

The anti-inflammatory activity of MFGM can be attributed to a combination of its components acting synergistically against various inflammatory factors. Palmano et al. demonstrated that complex phospholipid fractions of MFGM effectively alleviated delayed-onset foot and ankle swelling, a characteristic of the chronic/acute rheumatoid arthritis rat model. In addition, MFGM was found to suppress inflammation in the brains of SAMP8 mice, an age-dependent neuroinflammation model, by significantly inhibiting the pro-inflammatory cytokine tumor necrosis factor α and increasing the expression of transforming growth factor β1 [37]. Moreover, enteral supplementation of MFGM has been shown to alleviate short bowel syndrome-associated liver injury of Sprague Dawley rats by inhibiting the activity of the autophagy–inflammasome pathway [38]. The mechanisms of the protective effects are thought to be mediated by inhibiting NLRP3 inflammasome activation through MUC1 and sphingolipids. Furthermore, the intravenous administration of milk fat globule epidermal growth factor 8, a component of MFGM, significantly attenuated liver inflammation and oxidative stress in a hepatic ischemia–reperfusion animal model [39].

### 3.3. Reduction in Infection Susceptibility

MFGM exhibits antimicrobial activities by selectively inhibiting the adherence of pathogens [40,41]. In vitro studies have demonstrated that MFGM inhibits the binding of rotavirus (RV) to cell membranes. Both whole buttermilk and cheese whey MFGM showed dose-dependent inhibitory effects against RV [42]. Additionally, Monaco et al. found that milk lectin and MFGM components enriched in whey protein lipid concentrate exhibited inhibitory effects against porcine and human rotavirus in vitro [43]. The inhibitory effect of heated and raw MFGM on the expression of virulence genes by Escherichia coli O157:H7 has also been reported, with a stronger effect observed with heated MFGM [44]. Furthermore, bovine MFGM contains various antimicrobial agents, including membrane glycoproteins like mucin, xanthine oxidase, lactadherin, and glycosylated sphingolipids, which may prevent pathogen adherence [45]. In a recent trial involving healthy adults challenged with diarrheagenic *E. coli*, a milk concentrate rich in natural bioactive phospholipids and sphingolipids from MFGM was found to positively impact resistance to attenuated diarrheagenic *E. coli* [46].

### 3.4. Other Biological Effects of MFGM

Multiple animal studies with products containing MFGM have revealed long-term anti-carcinogenic activity, lipid-lowering effects, and regulation of gut microbiota. Purified sphingolipids, such as sphingomyelin, have demonstrated protective effects against colon cancer in animal models. Diets containing MFGM have also been found to protect against colon cancer in Fischer-344 rats, possibly due to their high content of polar lipids, particularly sphingomyelin [47]. In addition, MFGM has been shown to suppress body weight gain induced by high-fat diet (HFD) and reduce the mass of white adipose tissue (WAT), accompanied by decreased adipocyte sizes [48]. The mechanisms of the protective effects are achieved by promoting the browning of inguinal WAT, as evidenced by the upregulation of the protein expression level of uncoupling protein 1 (UCP1) in HFD mice. Dietary supplementation with MFGM has been shown to alleviate colitis and liver injury in mice by preserving the mucosal barrier, inhibiting oxidative stress, and modulating the gut microbiota [49]. Severe, repeated, or chronic stress can lead to negative health outcomes, including disruptions of the sleep/wake cycle and gut microbial dysbiosis. A diet containing MFGM has been shown to enhance sleep quality in rats, which is associated with changes in the gut microbiota [50]. Table 2 provides an overview of the in vivo and in vitro evidence for the multiple beneficial effects of MFGM components. Notably, most potential health benefits attributed to MFGM have been shown in in vitro or animal models. Some RCT findings suggested that MFGM in infant formula milk powder exhibited potential advantages in infant neurodevelopment and immune systems, as detailed in Section 5.2.2. Furthermore, multiple randomized controlled trials (RCTs) revealed that MFGM supplementation in combination with regular exercise has been shown to improve skeletal muscle strength [51,52,53]. Intriguingly, another RCT revealed that MFGM supplementation in a high-saturated fat meal decreased the postprandial insulinaemic (insulin levels after meals) and related immune responses in obese adults [54]. However, further RCTs involving adults are still essential to establish a more comprehensive understanding of MFGM’s beneficial effects.

While numerous studies have highlighted the diverse benefits associated with MFGM, including fostering neural development, reducing infection susceptibility, and regulating metabolic homeostasis, the complexities of MFGM hinder a complete comprehension of its interaction with human bodies. Additionally, there is a lack of sufficient evidence to substantiate which components of the MFGM, at what dosage, are effective and how the metabolic processes within the human body contribute to its beneficial effects. The absence of this information not only poses challenges to the current utilization of MFGM-based products in the market but, more significantly, impedes a comprehensive understanding of treating related diseases. Therefore, future research should delve deeper into the mechanisms of MFGM to elucidate how it impacts neural development, immunity, and metabolic homeostasis, benefiting the development of MFGM-based products.

## 4. Separation and Purification of MFGM

### 4.1. Separation of Milk Fat Globule

**Figure 3:**
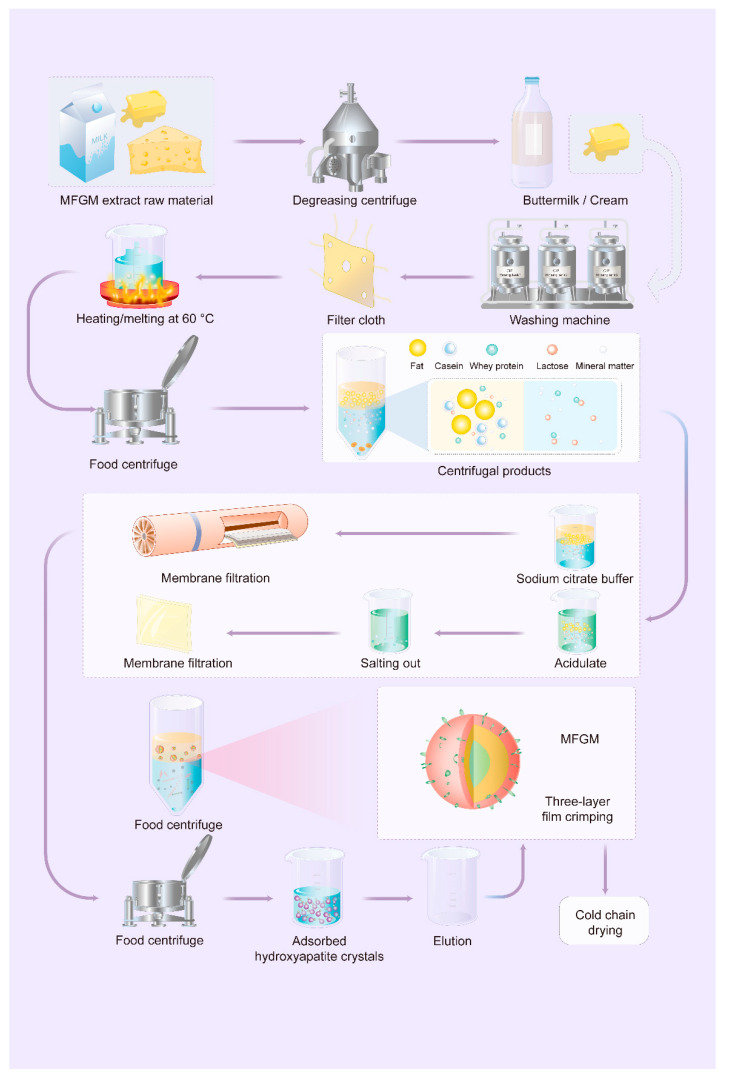
Schematic representation of the process for extracting MFGM from milk.

The isolation of MFGM can be divided into four steps: fat globule separation, cream washing, release of MFGM from the globules, and collection of the MFGM material [17] (Figure 3). When a pilot plant separator was used to fractionate raw milk at 55 °C, it yielded 30 to 35% milk fat cream and skim milk [17,58]. The cream obtained from separation was standardized to between 30 and 35% milk fat before further vat-pasteurized at 68.3 °C for 30 min. After cooling to 21.5 °C, the cream was tempered at 13 °C for 14 h for subsequent churning. Cream washing, which involves selectively removing MFGM components from the cream interface, may affect enzymatic activity. Different washing conditions have been employed, such as suspending the cream sample in water or various solutions without pH buffering, as well as a pH-buffered sucrose solution, phosphate-saline buffer, or simulated milk ultrafiltrate [59,60]. The cream-washing procedure was repeated twice more (Table 3). Buttermilk, collected via filtration through two cheesecloth layers, was used to retain minute butter granules. The obtained butter was melted at 60 °C after adding the same amount of deionized water. Finally, the butter was centrifuged in a laboratory centrifuge, and the MFG-containing serum or aqueous phase was recovered [61].

In addition to addressing the removal and effective utilization of non-fat globule membrane components, further exploration is needed to mitigate the impact factors, including pH value and temperature, in the industrial-scale separation and purification of MGFM.

### 4.2. Collection of MFGM

The membrane filtration of commercial butter serum can be used to isolate fractions rich in MFGM. The separation of MFGM from skim milk proteins in commercial buttermilk involves the addition of sodium citrate, followed by microfiltration through a 0.1 μm nominal pore size membrane. The use of sodium citrate facilitated the dissociation of casein micelles and allowed a large proportion of skim milk proteins to pass through the membrane [67]. This process effectively concentrated MFGM material in the retentate, demonstrating the potential of membrane filtration for producing MFGM fractions from commercial buttermilk [68]. Various filtration conditions, such as pH, temperature, pore size, and membrane material, have been investigated to improve membrane selectivity [69]. Other techniques that have also been used include cream washing, supercritical fluid extraction, acid precipitation of casein, the addition of rennet, and the use of chelating agents like citrate to dissociate casein micelles by chelating calcium, which have been suggested to enhance the efficiency of MFGM isolation [69]. However, implementing these methods on an industrial scale poses certain challenges. Recently, Iung et al. proposed a novel approach for separating MFGM from buttermilk using hydroxyapatite (HA) crystals. They found that MFGM isolates obtained from raw or pasteurized cream exhibited a high affinity for HA, suggesting the potential for improving MFGM valorization by separating it from buttermilk [70].

### 4.3. Extraction of Lipid and Protein from MFGM

**Figure 4:**
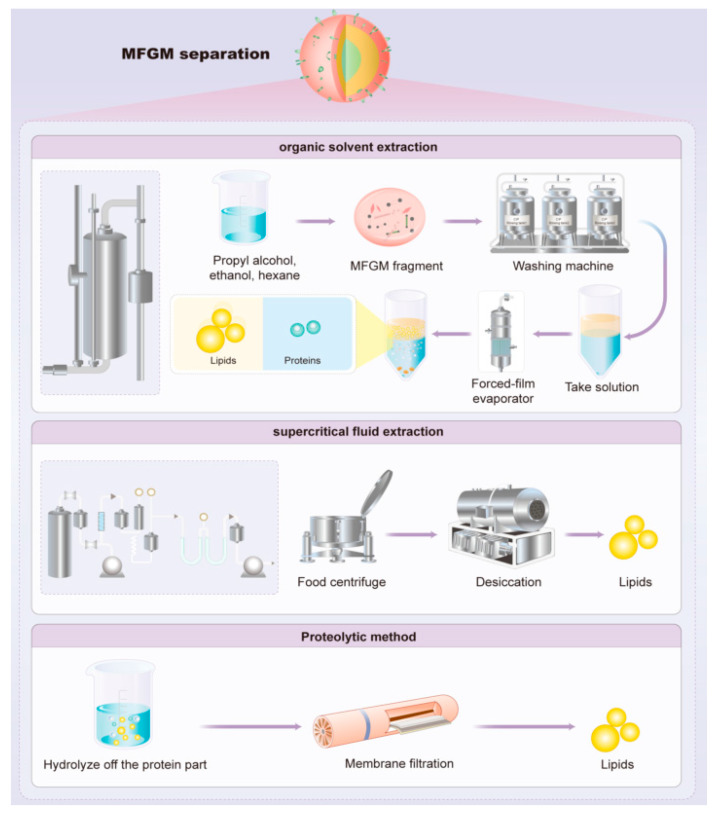
Schematic diagram depicting the isolation of lipid components from MFGM.

As shown in Figure 4, common methods for extracting polar lipids from MFGM include the following:

(1) Microfiltration and supercritical fluid extraction (SFE) were used to produce buttermilk-derived fractions with increased concentrations of polar MFGM lipids. A ceramic tubular membrane with a 0.8 μm pore size was employed to concentrate polar MFGM lipids, following a 2*n* factorial design. Subsequently, an SFE process utilizing supercritical carbon dioxide selectively removed non-polar lipid material from the microfiltered buttermilk product, resulting in a significant reduction in the non-polar lipid concentration along with a significant increase in the concentration of polar lipids from MFGM [71,72].
(2) Polar lipid concentrates can be obtained from milk processing by-products using organic solvent extraction. A mixture of hydrophobic and hydrophilic solvents (such as propanol or methanol) can effectively extract amphiphilic polar lipids, while hydrophobic solvents (such as hexane or chloroform) are required to extract triglycerides [73,74].

## 5. Interfacial Properties of MFGM and Its Applications in Food Industry

### 5.1. Surface Activity of MFGM

#### 5.1.1. Emulsifying Properties of MFGM

The emulsifying properties of MFGM are closely associated with its surface composition. The stability of oil-in-water emulsions relies on the surfactants present in the system. Both proteins and polar lipids possess emulsifying properties [75]. The hydrophilic and hydrophobic groups of proteins and lipids interact with the water and oil phases, respectively, reducing interfacial tension. MFGM can also impart a negative net charge to the surface of lipid droplets, preventing their aggregation through electrostatic repulsion. Additionally, the formation of micellar structures can enhance interfacial strength and improve the stability of fat droplets [75,76,77]. Furthermore, phospholipids can bind to proteins through hydrophobic and electrostatic interactions, leading to synergistic effects that improve the emulsifying properties and stability of emulsions [77]. Emulsions prepared using MFGM exhibit lower shear stress and display flow behavior similar to fluids, which can enhance their flowability [75].

#### 5.1.2. Effects of MFGM Proteins

**Figure 5:**
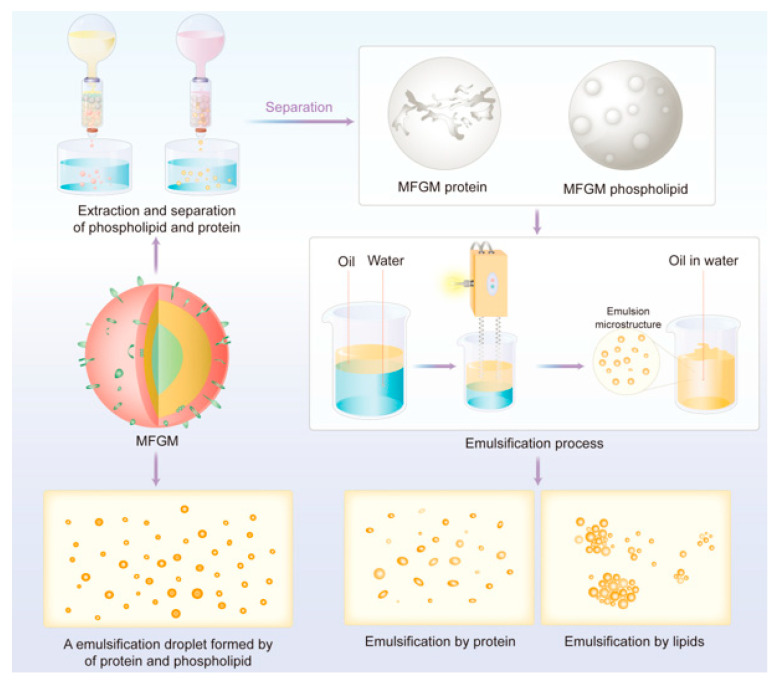
Schematic illustration of the liposomal encapsulation of drugs using MFGM lipids and their subsequent degradation in the gastrointestinal tract.

The role of MFGM proteins in stabilizing natural milk fat globules has been well-established [78]. The emulsion droplets formed by MFGM proteins are sensitive to pH and heat treatment, consistent with the behavior of proteins [75]. Proteins preferentially adsorb onto the surface of emulsion droplets, forming a viscous and elastic membrane layer that surrounds the oil droplets. This membrane generates electrostatic repulsion, preventing droplet aggregation [76]. Proteins play a crucial role in providing stability to emulsions. In an environment with a low concentration of MFGM proteins, lipid droplets lack adsorbed proteins, resulting in bridging flocculation and coalescence, leading to phase separation [75]. However, without the assistance of phospholipids, the formed protein membranes lack toughness and cannot maintain the stable dispersion of fat droplets (Figure 5) [79].

#### 5.1.3. Effects of MFGM Lipids

The phospholipids in MFGM, particularly phosphatidylcholine, can lower interfacial tension effectively and reduce the size of fat droplets significantly [3]. Priyanka, Sabine, Aman, Koen and Christophe [19] used compression isotherms to calculate the surface elasticity of MFGM components based on the Langmuir membrane balance method. The results demonstrated that polar lipids in MFGM possess higher surface elasticity, confirming their potential for stabilizing food systems. However, emulsion droplets prepared solely using MFGM proteins have smaller diameters than those prepared using the same concentration of MFGM phospholipids [75]. It has also been observed that using only lipids from MFGM as emulsifiers is not sufficiently effective [78,79]. This may be attributed to poor repulsion between the formed droplets, making them prone to reaggregation and flocculation.

### 5.2. Applications of MFGM

#### 5.2.1. Emulsifier

The primary purpose of adding emulsifiers to food is to enhance its texture and taste. Emulsifiers disperse oil and water evenly and prevent their later separation during storage, resulting in a finer texture, improved taste, and extended shelf life. Therefore, MFGM is highly valued for its nutritional value and emulsifying properties, making it a promising natural emulsifier alternative to commercial phospholipids, such as lecithin. However, excessive emulsifier usage can also have negative effects on human health. Jukkola et al., 2019, reported that in systems containing calcium and casein, such as milk powder, MFGM binds to calcium and was better able to inhibit calcium-induced casein flocculation than lecithin [5]. However, Yue Sun et al., 2023, argued that when MFGM is added to commercial formulations, it exists in a free form in the water phase, limiting its emulsification effect [4]. This may be due to the breakdown of MFGM in raw milk and a subsequent decrease in its functionality caused by homogenization. To address this, they introduced an additional structural lipid to simulate the enrichment of MFGM in infant formula. The experimental results indicated that compared to commercial infant formula, the addition of lipids created a microstructure with in vitro digestion of fat globules more similar to that of human milk. Similar conclusions were drawn by X. Yu et al., 2022, highlighting that the addition of phospholipids improves the emulsifying properties of MFGM by enhancing the interfacial composition of the emulsion, allowing for more effective maintenance of fat globule stability [80].

#### 5.2.2. Infant Formula Milk Powder

The addition of MFGM does not compromise food quality. MFGM promotes the digestion and absorption of lipids, as smaller lipid droplets have a larger surface area, facilitating faster digestion [81]. The emulsion droplets formed by MFGM are closer in size to those found in human milk, which reduces lipolysis and fat release in the stomach [4,9]. Furthermore, the pH-sensitive nature of MFGM proteins results in a significant decrease in their emulsifying ability in the presence of gastric acid. By incorporating MFGM into infant formula, the digestion rate in the gastrointestinal tract can resemble that of human milk [5]. Premature release of lipids in the stomach following the consumption of dairy products typically leads to the formation of large lipid lumps in the acidic environment, hindering absorption. Conversely, if lipids are released in the intestine, pancreatic enzymes hydrolyze it into fatty acids, which improves the absorption rate [82]. Additionally, the low content of polyunsaturated fatty acids in dairy phospholipids contributes to their superior oxidation stability compared to phospholipids from other sources [83]. Moreover, MFGM itself is rich in phospholipids and proteins, with proven nutritional value. These components have been associated with several positive effects of MFGM supplementation in infant formula, including better neurological development, gut health, and immune system function [9,83].

For instance, in a randomized controlled trial (RCT), infants fed an experimental low-energy, low-protein formula supplemented with bovine MFGM until 6 months of age demonstrated several positive outcomes, including improved performance in the cognitive domain of the Bayley Scales of Infant and Toddler Development 3rd Edition at 12 months of age [84]. Similarly, children who received MFGM-supplemented infant formula until 12 months of age exhibited enhanced cognitive outcomes in multiple domains at 5.5 years of age, including measures of intelligence and executive function [56]. Studies indicated that supplementation of formula with bovine MFGM can help bridge the cognitive development gap between breastfed infants and formula-fed infants [55,85,86,87]. Another RCT studied the outcomes of 240 infants who were fed standard formula (SF), bovine MFGM-supplemented experimental (EF) or breast milk (BF). The results demonstrated that the infants fed either BF or EF had substantially reduced incidence of acute otitis media (AOM) compared to the SF group. Moreover, supplementation with MFGM also led to a decrease in the use of antipyretics and modulated the humoral response to vaccine [88]. MFGM has beneficial regulatory effects on the gut microbiome, which led to it being used as a common ingredient in infant formula to enhance intestinal immunity and promote gut health [9]. The proteins, lipids, sugars, and other substances present in MFGM provide nutrients that support the growth of beneficial probiotic bacteria in the gut and have inhibitory effects against certain bacteria. In breastfed infants, *Bifidobacteria* and *Lactobacilli* dominate the gut microbiota [89], indicating that the addition of MFGM may promote the growth of these dominant taxa.

Despite the significance of MFGM, there is limited research on the content and proportions of substances in milk fat globule membrane (MFGM) in human milk, and examples for precise simulation of MFGM in infant formula are lacking. Thus, further exploration is still required to precisely adjust the composition of MFGM to simulate the components of human milk more accurately, which will be essential for the development of infant formula that closely mimics the nutritional composition of human milk.

#### 5.2.3. Other Applications

MFGM exhibits the potential to stabilize emulsions, foams, and suspensions due to its ability to form a film with good elasticity. The viscoelastic interfacial structure of the film provides resistance against mechanical disturbance, resulting in higher elasticity values [19]. The proteins present in MFGM contribute to the stabilization of the foam by forming a viscous and elastic network that relies on intermolecular interactions [76]. Le et al. successfully incorporated MFGM into yogurt, which improved its water-holding capacity and adhesiveness without affecting the fermentation process. Similarly, the addition of MFGM to dough can alter its rheological properties, significantly increasing the volume of bread loaves and preventing moisture loss in bread crumbs [12]. Furthermore, the high content of unsaturated fatty acids in MFGM opens up possibilities for developing antioxidants, while the high melting temperature of MFGM-derived phospholipids holds potential for various food and pharmaceutical applications [83]. MFGM also shows promise in enhancing the stirring performance of compound cream [6,11].

## 6. Encapsulation Systems with Lactoferrin as a Functional Ingredient

### 6.1. MFGM Liposomes

**Figure 6:**
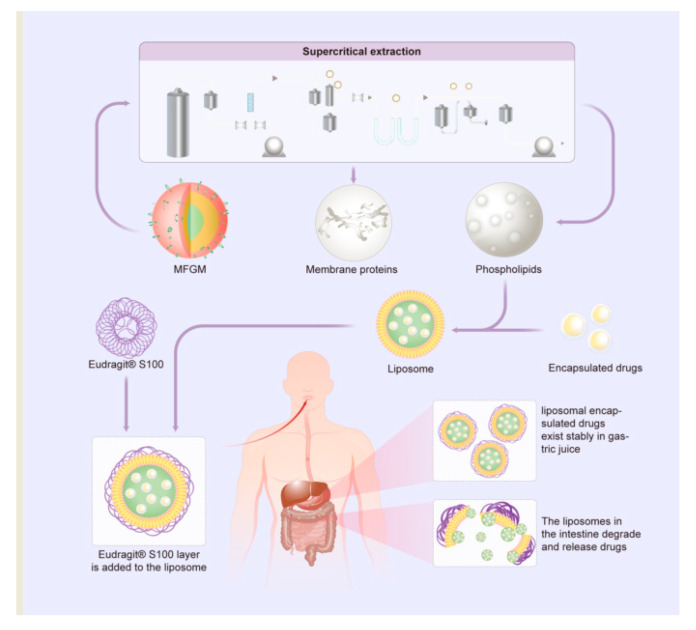
Microstructure of the emulsion observed under different conditions after the separate and combined participation of protein and lipid components from MFGM in emulsification.

Phospholipids constitute the second most abundant component of MFGM and possess amphiphilic properties arising from the presence of hydrophilic polar head groups and hydrophobic non-polar hydrocarbon tails [73,90]. When amphiphilic molecules come together, their hydrophobic tails/groups orient towards the inner layers, while their hydrophilic heads face the outer surface, resulting in the formation of double-layer spherical membrane structures known as liposomes [91]. The preparation of liposomes for food applications often involves techniques such as micro-fluidization, sonication, or membrane extrusion, with a preference for avoiding organic solvents [92]. Depending on the method employed, liposomes can consist of one or more bilayers, known as uni- or multilamellar liposomes, respectively. Their size ranges between 30 and 40 nm up to a few μm [93]. Due to their two-phase structure, lipid vesicles enable the entrapment, delivery, and release of water-soluble, lipid-soluble, and amphiphilic substances (Figure 6) [94,95].

### 6.2. Advantages of MFMG Liposomes as Encapsulation Materials

Compared to commonly used soy lecithin and egg yolk components, MFGM phospholipids offer several health advantages [96,97]. Liposomes derived from MFGM exhibit a high phase transition temperature, resulting in thicker membranes with lower permeability to mono- and divalent cations than those formed with non-hydrogenated soy phospholipids [98,99]. In a study comparing curcumin liposomes prepared with MFGM phospholipids and soy lecithin, it was found that under optimal conditions, the MFGM liposomes exhibited an encapsulation efficiency of approximately 74%, with an average particle size of 212.3 nm and ζ-potential of −48.60 mV. By contrast, the soy lecithin liposomes had an encapsulation efficiency of 63%, with an average particle size of 471.4 nm and ζ-potential of −7.63 mV. The MFGM liposomes displayed smaller particle size, higher absolute ζ-potential, and slower release rate compared to soy lecithin liposomes [80]. MFGM-derived phospholipids were also found to exhibit significantly higher entrapment efficiencies for β-carotene and potassium chromate [96].

Liposomes produced from soy phospholipids demonstrated a higher charge repulsion level than those derived from MFGM phospholipids, potentially enhancing their resistance against aggregation or coalescence between pH 6 and 7 [100]. Moreover, MFGM liposome dispersions exhibited superior stability during storage in the temperature range of 4–35 °C [99]. Soybean liposome dispersions were prone to faster aggregation and/or fusion under increased ion concentrations compared to MFGM liposome dispersions. Additionally, milk phospholipid liposomes demonstrated greater stability during storage and at different pH values compared to soy phospholipid liposomes [101]. The higher saturated phospholipid content and thicker membrane formation characteristics, attributed to liquid-ordered domains, likely contributed to the enhanced encapsulation and physical stability behavior of milk nanoliposomes compared to soy lecithin liposomes [101].

### 6.3. Application of MFGM Liposomes in Medicine

The selection of specific emulsifiers for infant formula can regulate the rate of lipid absorption to meet the nutritional needs of newborns by modulating intestinal lipolysis. A study demonstrated the influence of phospholipids in the surface layer of emulsion droplets on the activity of gastric lipase, a key enzyme involved in lipid absorption in neonates. Modifying the phospholipid composition enhanced gastric lipase activity, which in turn increased subsequent pancreatic lipase activity in the duodenum. This suggests the possibility of designing the surface layer of lipid droplets in infant formulas to maximize gastric lipase activity and improve fatty acid absorption in formula-fed neonates [102].

MFGM liposomes with an optimized chitosan coating hold promise as a potential delivery system for protein hydrolysates. A nanoencapsulation strategy employed chitosan-coated liposomes prepared with MFGM phospholipids to encapsulate antidiabetic peptides derived from Atlantic salmon (*Salmo salar*) protein hydrolyzate (SPH). The chitosan coating significantly improved the stability of the liposomes, with the best encapsulation efficiency (71.3%) and physical stability achieved using 10% MFGM phospholipid and 0.4% chitosan. Moreover, chitosan coating prolonged the release of SPH in simulated biological fluids [103].

A green, industrial-scale synthesis method has been developed for multivitamin-loaded and surface-modified liposomal microcapsules designed for site-specific intestinal delivery. These liposomes, tailored for pH-triggered delivery of lipophilic and hydrophilic pharmaceuticals, were synthesized using a mixture of MFGM phospholipids obtained from supercritical extraction of buttermilk powder. The liposomes were fabricated using an innovative organic solvent-free process based on the venturi-based rapid expansion of a supercritical solution. The liposomes were then coated with Eudragit^®^ S100 (Evonik (Piscataway, NY, USA)), a commercially available pH-responsive polymer, to protect the encapsulated payload from the gastric environment and enable pH-triggered release under simulated intestinal conditions. Their ability to co-encapsulate lipophilic and hydrophilic cargos while maintaining heat stability makes them highly suitable for effective oral delivery of bioactive compounds in pharmaceutical and food applications [104].

### 6.4. Application of MFGM Liposomes in Food Industry

The functionalization of MFGM-based nanostructures (NS) with antimicrobial biosurfactants holds promise for inhibiting the growth of cheese-associated pathogens, thereby enhancing the shelf life and safety of dairy products. Nisin-loaded rhamnolipid-MFGM-NS demonstrated potential applications as food-grade nano-antimicrobials and promising bioactive additives for the sustained preservation of cheese [105].

Nanoliposomes containing olive leaf phenolics have the potential to enhance the nutritional properties and commercial value of foods such as yogurt. Tavakoli et al. achieved high encapsulation efficiency (70.7–88.2%) and particle sizes ranging between 25 and 158 nm when incorporating olive leaf phenolics into nanoliposomes. The addition of these nanoliposomes to yogurt improved its antioxidant activity compared to normal yogurt, without affecting its color or sensory properties while reducing the syneresis rate [106].

The stabilizing effect of liposome encapsulation could be employed to protect vitamin C nutritional supplements in food products. Ascorbic acid, widely used as a vitamin supplement and antioxidant in food, was encapsulated with high efficiency inside liposomes, enhancing its stability compared to free aqueous solutions. This stability was particularly evident in the presence of common factors found in foods that typically contribute to its rapid degradation [107].

## 7. Conclusions and Future Trends

MFGM, particularly its polar lipid fraction, possesses significant nutritional values and is commonly used as an ingredient and emulsifier in infant formula to support the development of the neural and gut systems of neonates. Additionally, its unique physicochemical properties have facilitated the development of natural food additives and packaging materials. As efforts are being made to align the composition of infant formulas with that of human milk, various bioactive and biotic components are being incorporated to influence gut microbial composition and neurodevelopment [108], and MFGM holds tremendous potential in this regard. However, there are still many components and specific functions of MFGM that remain unclear. Further research to elucidate its mechanisms of action and refine the isolation and purification techniques will contribute to its broader and more extensive applications. Obtaining MFGM from defatted bovine milk poses challenges as the separation process is laborious while often also compromising its biological activity and structural integrity, thus impeding its commercialization [59,109]. Overcoming these limitations would position MFGM in various applications. The study of MFGM can enhance our understanding of its nutritional composition and functions, providing insights for the design of scientifically balanced diets. Furthermore, investigating the bioactive substances present in MFGM can provide new ideas for the research and development of healthy foods and medicines. In conclusion, the diverse bioactive substances derived from MFGM and their mechanisms of action hold significant implications for scientific research and human health.

## Figures and Tables

**Table 1:** Summary of representative proteins in MFGM and their functions.

Protein	Function	Purification Method	Molecular Weight (kDa)	References
Butyrophilin	Modulate MFG production and secretion; Might support the infant immune system.	Reversed-phase chromatography	66–67	[21]
Glycoprotein 2	Anti-adhesion and immunoprotective	Sepharose CL-4B gel filtration and cesium chloride density-gradient centrifugation	160–200	[22]
Xanthine oxidase	Redox reaction; anti-inflammatory	Sulfate fractionation and affinity chromatography	146–155	[23]
Glycosylation-dependent cell adhesion molecule 1	Mucin-like antibacterial component	Not be established	18–34	[24]
Carboxyl ester lipase	Hydrolyzes triglycerides, diglycerides, monoglycerides, vitamin A esters, cholesterol, and lysophospholipids in the gut lumen	Reverse-phase high-performance liquid chromatography	70–80	[25]
Lactoperoxidase	Catalyze oxidation of certain molecules and antimicrobial activity	Ammonium sulfate precipitation and ion-exchange chromatography	70–80	[26]
Lactoferrin	Promote probiotic growth and antimicrobial activity	Ion-exchange chromatography	70–80	[27]
Milk fat globule-EGF factor 8	Maintenance of intestinal epithelial homeostasis and the promotion of mucosal healing and phospholipid binding	Ammonium sulfate precipitation and immunoaffinity chromatography	47–59	[28]
Fatty acid-binding protein	Lipid transport and regulation of lipid metabolism	Not be established	13–15	[29]
Clusterin/ apolipoprotein J	Regulate apoptosis and protect other proteins from damage	Not be established	40–60	[30]
Perilipin 2	Protect lipid droplets from enzymatic attack by lipases	ion-exchange chromatography	45–55	[31]
Cluster of differentiation	Mediates innate immunity	immunoaffinity chromatography	76–78	[32]

**Table 2:** In vivo and in vitro evidence for the multiple functions of MFGM ingredients.

Ingredient Description	Model	Design	Primary Finding	Function or Activity	Reference
MFGM	Male BALB/c mice (7–8 weeks)	Intragastric gavage once a day for 18 weeks	Dietary supplementation by MFGM significantly improved memory and cognitive impairment	Neurodevelopment	[36]
Supplementation of formula with MFGM	Infants	Infants were randomized to receive either the experimental formula or SF from inclusion until 6 months of age	Higher concentrations of many phosphatidylcholines and altered concentrations of sphingomyelins	Neurodevelopment	[55]
Similar formula (milk fat globule membrane plus lactoferrin [MFGM + LF])	5.5 years of age in children	Added sources of bovine MFGM and bovine lactoferrin (bLF) through 12 months of age.	MFGM + LF improved cognitive outcomes in multiple domains at 5.5 years of age, including measures of intelligence and executive function	Neurodevelopment	[56]
MFGM complex lipid products	Rats, LPS-stimulated monocytes	MFGM preparations at 38.3 mg/g of diet (treatment groups) for two weeks, anti-arthritic drug via oral gavage (0.12 mg meloxicam/kg body weight (Metacam^®^, Boehringer-Ingelheim, St. Joseph, MO, USA))	MFGM to influence the activity and recruitment of inflammatory mediators	Anti-inflammatory	[57]
MFGM-10 Lacprodan	Five-week-old male Sprague Dawley (SD) rats	1.5 g/kg/d of MFGM for 15 days	Inhibition of NLRP3 inflammasome activation	Anti-inflammatory	[38]
Milk fat globule-EGF factor VIII (MFG-E8)	Male Sprague-Dawley rats (275–325 g)	rhMFG-E8; 160 μg/kg in 1 mL normal saline	Hepatic I/R induces significant accumulation of apoptotic cells in the live, modulation of PPARγ/NF-κB pathway	Anti-inflammatory	[39]
Buttermilk MFGM	MA-104 cells	125 μL containing the desired amount of MFGM fraction in MEM or MEM alone was mixed with an equal volume of RV containing approximately 1000 FFU of infectious activity.	MFGM inhibited the infectivity of the neuraminidase-sensitive OSU-RV strain in a dose-dependent manner	Anti-RV infection	[42].
MFGM	*E. coli* O157:H7	The resulting cell suspensions were diluted 1:100 in fresh medium with and without supplementation with MFGM. The MFGM was tested at 3 protein concentrations: 10, 100, and 1000 μg/mL	MFGM decreased the expression of *E. coli* O157:H7 virulence gene	Against the *E. coli* O157:H7	[44]
MFGM	Fischer-344 rats	a combination of AMF and MFGM. After a 7-day acclimation period on standard chow diets	MFGM intake can affect carcinogenesis	Against colon cancer	[47]
Whey-derived MFGM (MFGM-10 Lacprodan)	Male C57BL/6J mice (5-week-old)	High-fat diet with MFGM at 100 mg/kg BW, 200 mg/kg BW and 400 mg/kg BW for another 8-week	MFGM suppressed the protein and mRNA expression of PPARγ, C/EBPα and SREBP-1c and activated the AMPK pathway, correlating with the suppression of adipogenic differentiation. MFGM also induced the formation of brown-like adipocytes, as indicated by the upregulation of the protein expression of UCP1.	Anti-obesity	[48]
MFGM tablets (1 g MFGM/day)	Middle-aged Japanese adults	During the 10-week study period, the participants took either MFGM (1 g/day) or placebo (1 g/day of whole milk powder) tablets daily and participated in a bi-weekly exercise training program	Daily consumption of MFGM combined with regular exercise improve physical performance in healthy middle-aged adults	Improved physical performance, such as agility	[51]
MFGM tablets (1 g MFGM/day)	22 Japanese participants aged 60–73 years old	One group received MFGM tablets, while the other received placebo tablets daily for a duration of 10 weeks. Both groups participated in a light exercise program twice a week.	Daily supplementation with MFGM led to enhanced motor unit action potential conduction, resulting in improved muscle strength and physical performance in healthy Japanese adults aged over 60 years, who were also involved in bi-weekly light exercise.	Improved physical performance	[52]
MFGM tablets	115 middle-aged subjects (range 50–70 years old)	Six MGFM or Placebo tablets were taken by the participants per d for 24 weeks after breakfast	MFGM exhibited positive effects on the physical performance of community-dwelling Japanese adults	Improved physical performance	[53]
Dairy fraction rich in MFGM	Overweight and obese men and women (between 18 and 65 years of age)	Two isoenergetic high-fat meals. One meal includes a smoothie enriched with PO, while the other contains the same smoothie but without a cream-derived complex milk lipid fraction, which is a dairy fraction rich in MFGM	Addition of a dairy fraction rich in MFGM to a high-saturated fat meal reduced the postprandial insulinaemic and inflammatory response	Reduces the postprandial insulinaemic and inflammatory response	[54]

**Table 3:** Overview of various washing conditions using creams separators.

Type of Washing Solution	Number of Washing Steps	Cream Washing Solution	Temperature	References
Distilled or deionized water	8	1: 3	40 °C	[60]
0.25 M sucrose solution containing 2 mM MgCl_2_	4	1: 3	35 °C	[62]
0.25 M sucrose and 10 mM imidaxole (pH 7.1)	2	1: 1	15 °C	[63]
Phosphate buffer (NaH_2_PO_4_/Na_2_HPO_4_ 0.01 M, pH 7.2, 0.09% NaCl)	3	None	40 °C	[64]
Sucrose–saline solution (WS 1, WS 2, and WS 3)	3	None	None	[65]
Simulated milk ultrafiltrate containing 6 mol·L^−1^ urea and 50 mmol·L^−1^ EDTA	2	1: 10	20–25 °C	[66]

## Abbreviations

Milk fat globule membrane	MFGM
Mucin 1	MUC1
Xanthine dehydrogenase/oxidase	XDH/XO
Butyrophilin	BTN
Cluster of differentiation 36	CD36
Periodic acid–Schiff 6/7	PAS 6/7
Periodic acid–Schiff III, PAS III	also called MUC15
Adipophilin	ADPH
Fatty acid-binding protein	FABP
Glycosylation-dependent cell adhesion molecule 1	GlyCAM1
Phosphatidylethanolamine	PE
Phosphatidylcholine	PC
Phosphatidylserine	PS
Phosphatidylinositol	PI
Lysophosphatidylcholine	LPC
Sphingomyelin	SM
Phospholipids	PLs
White adipose tissue	WAT
High-fat diet	HFD
Supercritical fluid extraction	SFE
High-pressure homogenisation	HPH
